# 

**Published:** 1891-05

**Authors:** 


### CONTENTS—MAY, 1891.—VOL. VI., NO. 11.

SOCIETY NOTES—

Synopsis of Proceedings Texas State Med. Ass'n . . . . 457

Austin District Medical Society . . . . . . Under Contents.

EDITORIAL—

Vale. . . . . . . . . . . . . . . . . . . . . . . . 471

The Recent Meeting T. S. M. A. . . . . . . . . . . . . 474

MEDICAL NEWS AND MISCELLANY—

A very great number and variety of topics, of interest to readers . . . . . . . . . . . . . . . . . . . . . . 477

BOOK NOTICES—

About twenty-five books reviewed or noticed . . . . . . 484

PUBLISHER'S NOTES—

Concerning articles advertised . . . . . . . . . . . . . 489

